# Curriculum Design and Implementation of the Emergency Medicine Chief Resident Incubator

**DOI:** 10.7759/cureus.2223

**Published:** 2018-02-24

**Authors:** Michael A Gisondi, Adaira Chou, Nikita Joshi, Margaret K Sheehy, Fareen Zaver, Teresa M Chan, Jeffrey Riddell, Derek P Sifford, Michelle Lin

**Affiliations:** 1 Emergency Medicine, Stanford University School of Medicine; 2 Emergency Medicine, Brigham & Women’s Hospital, Harvard Medical School; 3 Emergency Medicine, Massachusetts General Hospital, Harvard Medical School; 4 Emergency Medicine, University of Calgary, Calgary, Alberta; 5 Faculty of Health Sciences, Department of Medicine, Division of Emergency Medicine, McMaster University; 6 Emergency Medicine, Keck School of Medicine of the University of Southern California, Los Angeles, CA; 7 Computer Science, Wayne State; 8 Department of Emergency Medicine, UCSF School of Medicine

**Keywords:** education, medicine, mentorship, resident, chief resident, social media, community of practice, online, digital, scholarship

## Abstract

Background

Chief residents receive minimal formal training in preparation for their administrative responsibilities. There is a lack of professional development programs specifically designed for chief residents.

Objective

In 2015, Academic Life in Emergency Medicine designed and implemented an annual, year-long, training program and virtual community of practice for chief residents in emergency medicine (EM). This study describes the curriculum design process and reports measures of learner engagement during the first two cycles of the curriculum.

Methods

Kern’s Six-Step Approach for curriculum development informed key decisions in the design and implementation of the Chief Resident Incubator. The resultant curriculum was created using constructivist social learning theory, with specific objectives that emphasized the needs for a virtual community of practice, longitudinal content delivery, mentorship for participants, and the facilitation of multicenter digital scholarship. The 12-month curriculum included 11 key administrative or professional development domains, delivered using a combination of digital communications platforms. Primary outcomes measures included markers of learner engagement with the online curriculum, recognized as modified Kirkpatrick Level One outcomes for digital learning.

Results

An average of 206 chief residents annually enrolled in the first two years of the curriculum, with an overall participation by 33% (75/227) of the allopathic EM residency programs in the United States (U.S.). There was a high level of learner engagement, with an average 13,414 messages posted per year. There were also 42 small group teaching sessions held online, which included 39 faculty and 149 chief residents. The monthly e-newsletter had a 50.7% open rate. Digital scholarship totaled 23 online publications in two years, with 67 chief resident co-authors and 21 faculty co-authors.

Conclusions

The Chief Resident Incubator is a virtual community of practice that provides longitudinal training and mentorship for EM chief residents. This incubator conceptual framework may be used to design similar professional development curricula across various health professions using an online digital platform.

## Introduction

In 2016, there were 10,119 residency training programs in the United States (U.S.) accredited by the Accreditation Council for Graduate Medical Education (ACGME). Most of these residency programs designate one or more of their senior residents as ‘chief residents,’ a one-year administrative designation with variable teaching and supervisory responsibilities [1-2]. Chief residents are commonly selected for the role because of outstanding clinical performance in their first years of training [3]. However, the duties of the chief resident are largely non-clinical. Chief residents are often expected to manage resident clinical schedules, arrange didactic conference curricula, resolve conflicts among peers, and address issues of resident morale and wellness [2]. These job expectations must be fulfilled while also advancing their own professional development and continued clinical training [4].

Chief residents who receive formal training for the role are better prepared to perform the assigned duties compared to those who do not [5]. Unfortunately, there are few opportunities for such training [1, 6]. Most commonly, residency directors send their chief residents to national conferences for specialty-specific, brief training courses. The ACGME is one of several organizations that offer interdisciplinary training for chief residents; however, like national meetings, these courses occur over just a few days and do not provide longitudinal training or mentorship for their participants [7].

In this paper, we describe the curriculum design and implementation of the Chief Resident Incubator, an annual, 12-month training program and virtual community of practice for chief residents in emergency medicine (EM). Outcome measures in this study reflect learner engagement during the first two cycles of this novel curriculum.

## Materials and methods

The design and implementation of the Chief Resident Incubator was informed by Kern’s Six-Step Approach for curriculum development for medical education, which includes problem identification, needs assessment for targeted learners, objectives, educational strategies, implementation, and program evaluation. The following summary of each step in the design and implementation process serves as the methodology for this curriculum project [8].

1. Problem identification

Most of the 227 EM residency programs in the U.S. select at least two senior residents to serve in the administrative role of chief resident; assuming a conservative minimum of two chief residents per program, there are an estimated 454 EM chief residents [9]. These individuals constitute our target population of potential learners. EM chief residents generally lack pertinent previous work experience and receive minimal training at their institutions to prepare them for the administrative duties of the role.

2. Needs assessment

The study authors performed an informal needs assessment for EM chief residents. Two specialty organizations in EM sponsor chief resident-specific tracks at their national meetings, each lasting one to two days [10-11]. Additionally, the ACGME Multi-Specialty and Pediatric Leadership Skills Training Programs for Chief Residents lasts five days and is available to senior residents from any specialty training program. There were neither online nor longitudinal training programs for EM chief residents in the U.S. prior to the launch of this curriculum.

In May 2015, Academic Life in Emergency Medicine (ALiEM) launched the Chief Resident Incubator in order to provide job training and professional development for chief residents in EM [12]. ALiEM (https://aliem.com) is a not-for-profit, digital health professions education organization that focuses on providing quality clinical content, developing collaborative resources, and sustaining online communities for EM practitioners. The study authors are all affiliated with ALiEM and receive a small annual stipend for their teaching and adminstrative efforts in the Chief Resident Incubator.

3. Objectives

The study authors generated goals and objectives for the curriculum through iterative discussions informed by a literature review and expert opinion. The four curricular objectives of the Chief Resident Incubator are (1) to foster a virtual community of practice for EM chief residents, (2) to deliver a longitudinal curriculum in administration and professional development relevant to the role of a chief resident, (3) to provide mentorship for participants, and (4) to facilitate multicenter scholarship among participants [13].

4. Educational strategies

Two conceptual frameworks informed the design of this curriculum. First, we applied the lens of Vyogtsky’s constructivist social learning theory to create our virtual community of practice, with a goal of developing an online learning environment that encourages collaboration, co-creation, and group learning [14]. The constructivist theory recognizes the social nature of learning as essential to effective learning and values contributions made by all participants of a community [14]. Chief residents are often divided as middle managers between their peers and faculty members [15]. It is important to create a community of practice for chief residents that allowed them to frequently interact with one another, thereby normalizing their experiences in the role and providing relevance to the curriculum content.

Second, we incorporated elements from Dube’s Virtual Community of Practice framework to create the program’s infrastructure [13, 16]. The Chief Resident Incubator is a temporary community, in that the membership was defined by the period in which we aimed to provide a one-year curriculum; this was matched to the common one-year timeframe of a chief resident year. The membership was limited to a peer group of learners at a similar stage of training. Participation was voluntary through enrollment in our closed educational platform. The geographic distribution of learners resembles that of virtually distributed networks commonly seen in global companies. Program faculty were intentional in their stewardship of the virtual community, through weekly facilitation of online discussions to promote learner engagement.

The word ‘incubator’ was deliberately chosen to reflect our instructional approach [17]. In business, an incubator is defined as a large initial investment in mentorship, capital, and networking for entrepreneurs, with goals to scale growth and ensure early success. This model translates well to the successful development of early academicians. Preparing our senior trainees to assume early leadership roles in healthcare is critical, as medicine and medical education continue to increase in complexity [18]. The chief resident role has traditionally been regarded as an entry-level leadership position that can launch the career of future academic faculty members [15]. For these reasons, there has been increasing interest in finding ways to ensure the early career success of chief residents [1, 7]. The Chief Resident Incubator provides continuous mentorship, programmatic resources, and a virtual network of peers to promote success in the chief resident role. Similar to business incubators, the approach of our Incubator Leadership Team to mentorship, team building, and learner engagement constantly evolves to meet chief resident needs and programmatic goals.

5. Implementation

The Incubator Leadership Team directs the curriculum and actively facilitates participation of the chief residents. This team includes a Chief Operating Officer, Chief Strategy Officer, the ALiEM Editor-in-Chief, and several EM faculty advisors who regularly execute initiatives within the curriculum.

A virtual community of practice benefits from the involvement of coaches, especially in its early, developmental stages [16]. For this reason, we annually invite ‘Virtual Mentors’ to serve each as month-long faculty experts and coaches. Virtual Mentors are selected based on their national reputation and scholarly expertise in selected curricular content, as well as their influential presence within medical education and social media. Additionally, we feature ‘insiders’ as guest faculty mentors; these individuals are exemplary physician-leaders who participate in one-time teaching sessions about leadership development and professional identity formation. For e.g., Dr. Richard Carmona (the 17th U.S. Surgeon General) and Dr. Mel Herbert (founder of a popular educational podcast series, EM: Reviews and Perspectives [19]) both served as 'insiders' in the first year of the curriculum. Together, the Incubator Leadership Team, Virtual Mentors, and Insiders comprise the program faculty.

The Chief Resident Incubator requires a significant commitment of faculty time. Collectively, members of the Incubator Leadership Team each document between 100-500 hours of effort annually; consequently, small honoraria are awarded from ALiEM and its industry sponsors.

The Incubator Leadership Team initially identified 11 topics to be delivered in a year-long curriculum, one per month, plus a four-week orientation to the program (Table 1). Each of the 12 Virtual Mentors are assigned to facilitate one of the monthly topics, delivered using just-in-time teaching [20].

**Table 1:** Chief Resident Incubator: curriculum

Month	Topic
May	Orientation
June	What Makes a Leader?
July	Negotiation Skills
August	Best Practices of Clinical Scheduling
September	Bedside Teaching
October	Interviewing Skills
November	Building a Personal Brand
December	Project Management
January	Learning from Mistakes
February	Middle Management
March	Working with Difficult Residents
April	Physician Wellness

In addition to the monthly curriculum, the Incubator Leadership Team offers special events throughout the year, including online book clubs, a financial advice series, and the 'Insiders' seminar series. All presentations are conducted, recorded, and archived as a video, using Google Hangout on Air (Google Inc., Mountain View, CA on YouTube Live, https://youtube.com). Each recorded session includes a Virtual Mentor, at least one faculty member from the Incubator Leadership Team, and three to five participating chief residents; all participants provide verbal consent to be recorded.

We primarily use Slack (Slack Technologies, San Francisco, CA; https://slack.com), a virtual messaging and discussion platform, as the communication tool for Incubator faculty and participants. Slack discussions are summarized monthly by newsletter editors for the participants using an e-mail-based newsletter service, MailChimp (MailChimp, Atlanta, GA; https://mailchimp.com). The combined use of Slack, MailChimp, and Google Hangout on Air videos supplant the need for a traditional digital course management system. The technologies used in the Chief Resident Incubator are summarized in Table 2.

**Table 2:** Technologies used in the Chief Resident Incubator

Platform	Description	Use
Slack - Slack Technologies, San Francisco, CA.	Slack is a cloud-based, communication network that features a series of interconnected chat rooms, document storage, and integration with numerous third-party project management platforms.	Slack is the primary communication tool for faculty members and participants.
Google Hangouts on Air, hosted by YouTube Live - Google Inc., Mountain View, CA. Google Hangout on Air,	Google Hangouts on Air is a free, online communication platform that supports video chat among a group of individuals. Discussions may be recorded, live streamed, and archived on YouTube Live.	Small group discussions between faculty members and residents are recorded and archived using Hangouts on Air.
MailChimp - Rocket Science Group, Atlanta, GA.	MailChimp is an automated email marketing platform.	This platform is used for the creation and distribution of a monthly e-newsletter.
Academic Life in Emergency Medicine (ALiEM).	ALiEM is a not-for-profit, digital health professions education organization that hosts an educational blog, sponsors virtual communities of practice, and conducts scholarship.	The ALiEM blog is used to disseminate scholarship and other educational content developed by the program participants.

In addition to online lectures and discussions, the chief residents are invited to two in-person sessions, each lasting two hours. These sessions coincide with two major EM national conferences that are often attended by chief residents: a program launch event at the Society of Academic Emergency Medicine Annual Meeting in May, and a mid-curriculum event at the American College of Emergency Physicians Scientific Assembly in October. These sessions offer an opportunity for course faculty and participants to interact in-person, ultimately enhancing our online community through a culture of psychological safety, trust, and cohesiveness for the remainder of the year.

Professional development opportunities are also made available to the chief residents, such as the Visiting Chief Resident Grand Rounds Speaker series, educational grants, and mentored blog publications. First, chief residents can apply to present as an invited guest lecturer at Grand Rounds by submitting video vignettes of their teaching. Those selected have been sponsored to lecture at such institutions as Brigham and Women’s Hospital, New York University-Bellevue Hospital, Northwestern University, and University of California San Francisco. Chief residents selected for these opportunities receive feedback on slide design and public speaking skills by members of the Incubator Leadership Team before their presentations. Second, there are educational grants available to participants. For e.g., EBSCO Health/DynaMed Plus© awarded a wellness grant to support residency-based wellness initiatives to one residency program and its chief residents. And third, interested chief residents are provided the opportunity to receive mentorship in publishing on an educational blog website, which receives 1.5 million annual page views.

Programmatic expenses include books provided to the chief residents during the curriculum, facility costs of the in-person events, and general administrative overhead. To finance the operating costs of the Chief Resident Incubator, residency programs support tuition in the program at $200 per chief resident. Furthermore, the Chief Resident Incubator is exclusively sponsored by an unrestricted educational grant from EBSCO Health/DynaMed Plus©.

6. Program evaluation

We report descriptive outcomes of the Chief Resident Incubator for the first two years of the curriculum, spanning the period of May 1, 2015 to April 30, 2017. Primary outcomes reflect modified Kirkpatrick Level One outcomes for digital learning: evaluation of reaction via online engagement [21]. The outcome measures include (a) enrollment, (b) Slack message activity, (c) Google Hangout on Air participation, (d) e-newsletter engagement, and (e) digital scholarship. This project received exempt status by the Stanford University Institutional Review Board.

## Results

Table 3 summarizes learner engagement for the first two years of the curriculum. An average of 206 chief residents from 75 residency programs enrolled annually in the Chief Resident Incubator from 2015-17, representing 33% of the allopathic EM residency programs in the U.S.

**Table 3:** Measures of learner engagement with the Chief Resident Incubator online curriculum, 2015-17

	May 2015 – April 2017	May 2016 – April 2017
ENROLLMENT		
# Chief residents	204	209
# Residency programs	73	77
SLACK MESSAGE ACTIVITY		
# Total messages	11,000	15,828
% Public messages	32	69
% Private group messages	15	9
% Direct messages	53	22
GOOGLE HANGOUTS ON AIR (GHOA)		
# GHOA small group presentations	21	21
# Faculty participants	15	21
# Chief resident participants	63	86
E-NEWSLETTER ENGAGEMENT		
% Click rate	59	42.4
DIGITAL SCHOLARSHIP		
# Total online publications	7	16
# Chief resident co-authors	28	39
# Faculty co-authors	3	18

The Slack platform hosted an annual average of 30 public discussion channels on topics related to chief resident administrative activities, such as conference, scheduling, educational projects, and wellness. In addition to public channels, learners created private channels for group messaging, as well as direct messages among participants used to faciliate projects and provide direct mentorship. An average of 13,414 messages were posted across two years: 50.5% as public channel posts, 12% as private group messages, and 37.5% as direct messages.

A total of 21 Google Hangout on Air presentations occurred annually, each of which was archived for review by all participants at their convenience. A total of 36 faculty members and 149 chief residents participated in the live broadcast of at least one of these Google Hangouts.

Monthly e-newsletters delivered to all Incubator members were opened by an average of 50.7% participants; for comparison, the industry average reported by MailChimp for education-related newsletters is 22% [22].

A bibliography of digital scholarship for each cycle of the curriculum is summarized in Table 4, totaling 23 online publications co-authored by 67 chief resident and 21 faculty members. As per ALiEM blog editorial standards, each of these items underwent a thorough peer review by one or more ALiEM editors using a previously published review process [23].

**Table 4:** Digital scholarship authored by participants of the Chief Resident Incubator, 2015-17

2015 – 2016
Joseph M, Stuart R, Paetow G, Wojtal N, Cohen V, Lindsey J, Gopalsami A, Schneberk T, Patel S, Sheehy M. [Blog Post] “Dear Residents: 10 Things Your New Chiefs Want You to Know.” ALiEM, 5/14/15.
Sheehy M, Harding A. “ALiEM Book Club: The White Coat Investor: A Doctor’s Guide to Personal Finance and Investing.” ALiEM, 8/14/15.
Shaikh S, Risler Z, Hansen M, Horan C, Burrup D, Harding A, Chou A. [Blog Post] “5 Scheduling Software Options in the Emergency Department: An In-Depth Review.” ALiEM, 8/27/15.
Stuart R. “ALiEM Book Club: Dreamland: The True Tale of America’s Opiate Epidemic.” ALiEM, 10/9/15.
Stuart R, Joshi N. “ALiEM Book Club: Bouncebacks! Emergency Department Cases: ED Returns.” ALiEM, 2/12/16.
Trop A, Zaver F, Gottlieb M, Glenn M. [Blog Post] “ALiEM Chief Resident Incubator Must Read EM Journal Articles - 2016 Edition.” ALiEM, 2/19/16.
Gottlieb M, Habrat D, Sheehy M, Zidovetzki S, Chou A, eds. [Online Textbook] ALiEM In-Training Exam Prep: Emergency Medicine, 1^st^ Edition. ALiEM Publishing: San Francisco, CA, 6/1/2016. ISBN 978-0-9907948-7-5
2016 – 2017
Rose C, Weir A. [Blog Post} “Top 5 FOAM Radiology Resources: ALiEM Chief Resident Incubator Recommendations.” ALiEM 6/9/16.
Craddick M, Giordano J, Gronowski T, Kalnow D, Pusateri M, Sanford A, Zaver F, Chou A. [Blog Post] “Top 10 Secrets to Success as an Emergency Medicine Resident.”ALiEM, 6/21/16.
Liu EL, Rose C, Dyer S, Zaver F, Chou A. [Blog Post] “A Starter’s Roadmap to EM Resources: Books, Websites, and Apps.” ALiEM, 8/3/16.
Gronowski T, Sanford A, MD, Liang L, Glenn M, Joshi N. “ALiEM Book Club: A Thousand Naked Strangers: A Paramedic’s Wild Ride to the Edge and Back.” ALiEM, 8/14/16.
Burkhardt J, Watsjold B, MD, Fan T, Dyer S. [Blog Post] “MDPV Card: Introduction to ED Charting and Coding.” ALiEM, 8/15/16
Dyer S, Burkhardt J, Watsjold B, Fan T, Trop A. [Blog Post] “MDED Charting and Coding: History of Present Illness & Past Medical, Family, Social History.” ALiEM, 9/5/16.
Pusateri M, Battaglioli N. [Blog Post] “10 Tips to Become a Successful Interviewer: Do’s and Don’ts.” ALiEM, 9/15/16.
Watsjold B, Dyer S, Burkhardt J, Trop A. [Blog Post] “MDED Charting and Coding: Review of Systems (ROS).” ALiEM, 11/2/16.
Fan T, Burkhardt J, Watsjold B, Trop A. [Blog Post] “MDED Charting and Coding: Physical Exam (PE).” ALiEM, 11/9/16.
Abubshait L, Gottlieb M, Haas M. [Blog Post] “MDPV Card: Hip Injuries | Quick Reference Guide.” ALiEM, 11/14/16.
Watsjold B, Dodd K, Trop A. [Blog Post] “MDED Charting and Coding: Medical Decision Making (MDM).” ALiEM, 11/16/16.
Abubshait L, Gottlieb M, Haas M. [Blog Post] “MDPV Card: Knee Injuries | Quick Reference Guide.” ALiEM, 11/21/16.
Gottlieb M, Zarzar R, Bierny P, eds. [Online Textbook] ALiEM In-Training Exam Prep: Emergency Medicine, 2nd Edition. ALiEM Publishing: San Francisco, CA, 11/25/2016. ISBN 978-0-9907948-8-2
Glenn M, Little A, Haas M. [Blog Post] “MDPV Card: Elbow Injuries.” ALiEM, 12/12/16.
Battaglioli N. [Blog Post] “Winners of the 2017 EBSCO Health/DynaMed Plus Wellness Grant.” ALiEM, 1/27/17.
Lee H, Abubshait L. [Blog Post] “MDPV Card: Laceration Repair and Sutures – A Cheat Sheet Guide.” ALiEM, 3/6/17.

## Discussion

We describe a unique, longitudinal training program that allows EM chief residents to connect and form a virtual community of practice. To our knowledge, it is also the only virtual community of practice for chief residents. Our results demonstrate active learner engagement and scholarly productivity, indicating that the design and implementation of such communities for resident trainees is valuable. Two years of experience with this unique curriculum has provided valuable insights into the nuances and dynamics of fostering such virtual learning teams.

The Chief Resident Incubator is administered using a variety of digital communications software to facilitate frequent and efficient interaction between learners, with a goal of fostering community and context. We observed that this effort was enhanced by in-person sessions, to promote engagement and trust. Our primary outcome measures for the evaluation of this curriculum were therefore chosen to reflect the degree of engagement and online interaction of our learners. [21]

We envision that virtual teams will become more popular in health professions education, as trainees, clinicians, and educators become more digitally proficient. Technology has already facilitated the work of “globally distributed teams” in research collaboration [24]. As with all new online courses, an orientation to the curriculum and its digital platforms was essential for early adoption and engagement. In particular, we observed that participants who had never used Slack were slower to engage with the curriculum than those already familiar with the platform.

Our experience with this curriculum suggests that new learning outcomes can be realized for teams of learners using the conceptual framework of a virtual community of practice. For example, we report a total of 23 digital publications by 76 chief resident co-authors over the first 24 months of the Chief Resident Incubator. These opportunities for digital scholarship, and the faculty oversight they required, could not have been possible in any other chief resident training platform to date. Our positive experiences in building a virtual community for resident learners informed our decision to further invest in this model of training for fellows (ALiEM Fellowship Incubator) and junior faculty (ALiEM Faculty Incubator).

EM as a specialty is extremely engaged in online and social media technologies for medical education [25-26]. It is likely that the assimilation of our faculty and chief residents to this new virtual community of practice resulted from familiarity with ALiEM, social media, digital learning, and the technologies that we used to deliver the curriculum. These technologies limited some scaling of our curriculum. For instance, Google Hangout on Air has a bandwidth that supports a limited number of participants, and the no-cost version of Slack limits the archiving of posts at 10,000 messages. To build similar incubators for larger learner groups would require additional financial resources for more costly, yet scalable online platforms.

There are several important limitations to our current program, its evaluation, or both. Importantly, we sought to monitor outcome measures of engagement with the curriculum by learners, as surrogate markers for the success of our virtual community of practice [21]. We have not yet studied the impact of our curriculum on the job performance of our chief residents, nor have we examined knowledge retention. Though we have anecdotal evidence that our learners enjoyed the format of a virtual community of practice, we have not formally measured their satisfaction with the curriculum. Future studies are needed to assess these learning outcomes.

The Chief Resident Incubator was scaled for a cohort of EM chief residents with a highly motivated group of expert facilitators. It may not hold true that other online communities will have similar successes, as these communities will vary based on the level of engagement of the facilitators and participants. Our Incubators are implemented by a geographically distributed team of academic emergency physicians who receive very small honoraria relative to the hundreds of hours of teaching effort they provide. It is unlikely that a competent volunteer model could be easily replicated.

## Conclusions

The Chief Resident Incubator is a longitudinal, virtual community of practice that provides administrative training, professional development, and mentorship for EM chief residents. The high level of curricular engagement by participants across the U.S. suggests that a digital platform can facilitate the development of unique communities of practice to address educational needs and foster scholarship.
